# Seasonal variation influences fibromyalgia severity in terms of widespread pain among female patients: data from a large national registry

**DOI:** 10.1007/s10067-025-07697-1

**Published:** 2025-09-22

**Authors:** Marco Di Carlo, Sonia Farah, Manuela Di Franco, Cristina Iannuccelli, Annunziata Capacci, Serena Guiducci, Giovanni Biasi, Roberto Giacomelli, Laura Bazzichi, Fabiola Atzeni, Piercarlo Sarzi-Puttini, Fausto Salaffi

**Affiliations:** 1 Department of Clinical and Molecular Sciences, Rheumatology Unit, Università Politecnica Delle Marche, Jesi, Ancona, Italy; 2 Department of Internal Clinical, Anesthesiologic and Cardiovascular Sciences, Rheumatology Unit, Sapienza University of Rome, Rome, Italy; 3 UOC Reumatologia, Fondazione Policlinico Universitario A. Gemelli IRCCS, Rome, Italy; 4 Department of Experimental and Clinical Medicine, Divisions of Internal Medicine and Rheumatology AOUC, University of Florence, Florence, Italy; 5 Department of Medical Sciences, Surgery and Neurosciences, Rheumatology Unit, University of Siena, Siena, Italy; 6 Clinical Unit of Immunorheumatology, Fondazione Policlinico Universitario Campus Bio-Medico, Via Alvaro del Portillo, 200, 00128 Rome, Italy; 7 Research Unit of Immunorheumatology, Department of Medicine and Surgery, Università Campus Bio-Medico Di Roma, Via Alvaro del Portillo, 21, 00128 Rome, Italy; 8 Rheumatology Unit, AOU Pisana, Pisa, Italy; 9 Rheumatology Unit, IRCCS Ospedale Galeazzi - S. Ambrogio, Università Degli Studi Di Milano, Milan, Italy; 10 Department of Internal Medicine, Rheumatology Unit, University of Messina, Messina, Italy

**Keywords:** Disease severity, FASmod, Fibromyalgia, PSD, Seasonality, WPI

## Abstract

**Introduction/objectives:**

Seasonal variation may influence musculoskeletal pain, and fibromyalgia is primarily characterized by widespread chronic pain. This study aimed to assess whether symptom severity in fibromyalgia varies by season.

**Methods:**

A retrospective, cross-sectional analysis was conducted on patients from the Italian Fibromyalgia Registry. Patients were grouped based on the season of their clinical evaluation. Disease severity was measured using disease-specific clinimetric tools: the Polysymptomatic Distress Scale (PSD), including the Widespread Pain Index (WPI) and Symptom Severity Scale (SSS), as well as the revised Fibromyalgia Impact Questionnaire (FIQR) and the modified Fibromyalgia Assessment Status (FASmod). Statistical analyses included the Kruskal–Wallis test and pairwise comparisons using the Dwass–Steel–Critchlow–Fligner test.

**Results:**

A total of 2614 patients were evaluated. Significant seasonal differences were found for both WPI (*p* = 0.042) and FASmod (*p* = 0.037). In female patients, these differences were more pronounced (WPI, *p* = 0.016; FASmod, *p* = 0.018), while no significant variation was observed in males. Pairwise analysis showed higher symptom severity in autumn compared to summer for both WPI (*W* = −4.009; *p* = 0.024) and FASmod (*W* = −3.800; *p* = 0.037).

**Conclusion:**

In fibromyalgia, widespread pain appears more severe in autumn than in summer, particularly among female patients. These findings highlight the potential role of seasonality in symptom modulation and underscore the importance of incorporating seasonal factors into patient management and education.

**Table:** 

**Key Points** • *Seasonality influences the severity of fibromyalgia in terms of widespread chronic pain*. • *Findings from a large national registry (2,614 patients) demonstrate significantly higher Widespread Pain Index and modified Fibromyalgia Assessment Status scores during the autumn months compared to the summer*. • *The increased severity of widespread pain in autumn is specifically observed among female patients*.	

## Introduction

Fibromyalgia is a condition characterized by a wide range of disabling symptoms, the most prominent of which include chronic widespread pain, fatigue, and non-restorative sleep, affecting approximately 5% of the general population [1, 2]. The pathophysiological mechanisms underlying fibromyalgia remain largely elusive, with no unified theory explaining its etiology [3–5].

The accurate clinical assessment of symptoms, on which the diagnosis is based, is challenging [6], as the manifestation of fibromyalgia can be influenced by numerous variables, some of which are external to the patient, and fibromyalgia is fully classified within chronic pain conditions that align with the so-called “biopsychosocial model” [7]. Recent studies have demonstrated, for instance, that geographical origin within the same country can influence the severity of fibromyalgia [8].

Among the potential external variables influencing the course of chronic pain, temporal patterns represent a relevant factor. The impact of such temporal dynamics on health conditions (and pathological states) is the subject of chronobiological research. It is well established, for instance, that chronic pain is substantially modulated by circadian rhythms. A recent systematic review of the literature, encompassing various chronic pain conditions with heterogeneous pathogenetic mechanisms, documented that fibromyalgia-related pain tends to be more severe in the morning, whereas neuropathic pain typically intensifies during the evening hours [9].

Seasonality refers to the intrinsic characteristic of a phenomenon to manifest periodic and cyclical fluctuations at regular, predetermined temporal intervals, typically associated with specific phases of the annual cycle, such as climatic seasons. Seasonality exerts significant effects on human health, a phenomenon particularly recognized in the epidemiology of certain infectious, psychiatric, and cardiovascular diseases [10]. Seasonality refers to chronobiology and, in the context of fibromyalgia, has been discussed for over 30 years, starting with a study by Moldofsky, which reported more pronounced seasonal variations in patients with fibromyalgia compared to those with rheumatoid arthritis, particularly in terms of affectivity and pain. In fibromyalgia patients, overall health status was found to be worse from November to March and better from May to August [11].

However, data on the impact of seasonality on fibromyalgia remain limited. A Spanish study conducted in 2023 reported no seasonal variations in symptom severity, assessed across multiple health domains, including pain intensity, fatigue, physical function, stiffness, anxiety, depression, psychological distress, and both the quality and quantity of sleep [12].

Starting from these considerations, the aim of the present study was to determine whether seasonality affects fibromyalgia severity using data from a large national database.

## Methods

### Setting

The study is a retrospective cross-sectional analysis of patients included in the Italian Fibromyalgia Registry (IFR) from November 2018 to January 2021. The IFR collects data from patients diagnosed with fibromyalgia who are referred to multiple rheumatology units across Italy [13]. Enrollment in the IFR does not imply any therapeutic intervention, as its purpose is purely clinical and clinimetric.

Inclusion in the IFR is based on fulfillment of the 2010/2011 American College of Rheumatology (ACR) criteria for fibromyalgia [14]. The diagnosis of fibromyalgia for inclusion in the IFR is based on the 2010/2011 ACR criteria rather than the subsequent 2016 ACR criteria, since the electronic platform was developed according to those criteria and, for reasons of diagnostic uniformity, they have been retained.

For the analyses conducted in this study, all patients with coexisting conditions that could interfere with clinimetric assessment were excluded. These included chronic inflammatory joint diseases or connective tissue diseases, neurodegenerative disorders, severe depression, uncontrolled endocrinopathies, and active malignancies.

The procedures conducted within the IFR were approved by the ethics committee of the reference center (Comitato Etico Unico Regionale [CERM], approval number 1970/AV2) and all patients included in the IFR provided informed consent for the anonymous collection of data.

### Assessment

The IFR allows for the collection of longitudinal data. For the analyses conducted in this study, each patient’s baseline evaluation was considered, which corresponds to the visit at the time of inclusion in the IFR.

The clinimetric evaluation to determine the severity of fibromyalgia was based on the PolySymptomatic Distress Scale (PSD), the revised Fibromyalgia Impact Questionnaire (FIQR), and the modified Fibromyalgia Assessment Status (FASmod). Each scale has specific characteristics.

### PSD

The PSD substantially corresponds to the 2010 ACR diagnostic criteria and subsequent modifications, which have revolutionized the interpretative framework of fibromyalgia. It is a fully patient-reported measure. The PSD is the arithmetic sum of the Widespread Pain Index (WPI) and the Symptom Severity Scale (SSS).

The WPI refers to the presence (score = 1) or absence (score = 0) of pain in 19 specified body regions, indicated on a front/back mannequin. The total WPI score ranges from 0 to 19.

The SSS assesses the severity of non-pain symptoms such as fatigue, memory problems, and unrefreshing sleep using numerical scales from 0 to 3 (where 0 indicates no problem and 3 indicates a severe problem). Additionally, the presence (score = 1) or absence (score = 0) of abdominal pain, depression, or headache is included. The total SSS score ranges from 0 to 12.

Thus, the total PSD score ranges from 0 to 31 [14]. Beyond its diagnostic purpose, the PSD can also serve as an interpretative tool in evaluating the severity of fibromyalgia [15].

### FIQR

The FIQR includes 21 numeric rating scales, each ranging from 0 to 10, grouped into three health domains and referring to the patient’s experiences over the past 7 days.

Specifically, nine items assess the physical function domain, two items assess the overall health status domain, and ten items assess the symptoms domain. The total score ranges from 0 to 100, with higher scores indicating greater disease severity.

The final score is calculated as the weighted sum of the three domains: the physical function domain score is the sum of the nine items divided by three; the overall health status domain score is the unmodified sum of the two items; and the symptoms domain score is the sum of the ten items divided by two [16]. The Italian validated version of the FIQR has been used for the purposes of this study [17].

### FASmod

The FASmod is a simplified tool, a short version of the Fibromyalgia Assessment Status (FAS), which refers to symptoms experienced over the past seven days. The FASmod assesses the presence of fatigue and non-restorative sleep using two 11-point numerical rating scales, and the presence of widespread chronic pain using a body mannequin divided into 19 body areas (each painful area is scored as 1). The final score, ranging from 0 to 39, is the arithmetic  sum of the two numerical scales and the count of painful body areas on the mannequin [18].

### Statistical analysis

The data collected by each individual center participating in the IFR were exported to the coordinating center (Rheumatology Unit, Università Politecnica delle Marche).

To address the study’s primary objective—namely, to determine whether seasonal variation has an impact on the severity of fibromyalgia—patients were categorized into four groups based on the season (winter, spring, summer, autumn) of year at which they were enrolled in the IFR.

The normality of the data distribution was evaluated using the Shapiro–Wilk test. As the clinical data and scores from the clinimetric scales did not exhibit a normal distribution, non-parametric statistical analyses were applied and data are presented as median and interquartile range (IQR). The Kruskal–Wallis test was used to compare the four patient groups based on the season of evaluation. Post hoc pairwise comparisons between seasonal groups were conducted using the Dwass–Steel–Critchlow–Fligner (DSCF) test. For both tests, *p*-values < 0.05 were considered statistically significant. The analysis was conducted using Jamovi software, version 2.6.26.

## Results

### Demographic data and characteristics of the entire cohort

The study included a total of 2614 patients, comprising 2450 females and 164 males, with a median age of 57.00 years (IQR: 49.00–64.00) and a median body mass index (BMI) of 24.52 (IQR: 21.87–28.00).

Regarding disease severity indices, the median PSD score was 20.00 (IQR: 16.00–24.00), with a median WPI of 11.00 (IQR: 8.00–15.00), a SSS score of 9.00 (IQR: 7.00–10.00), a total FIQR score of 68.00 (IQR: 52.00–80.00), and a FASmod score of 28.00 (IQR: 22.00–32.00).

Complete cohort characteristics and assessments of normality distribution are provided in Table 1.

**Table 1:** Descriptive statistics of the main study variables and assessment of normal distribution

	Descriptive statistics				Shapiro–Wilk test	
Variables	Mean	SD	Median	IQR	*W*	*p*
Age (years)	56.29	12.56	57.00	49.00–64.00	0.985	< 0.001
BMI (Kg/m^2^)	25.46	5.15	24.52	21.87–28.00	0.926	< 0.001
FIQR total	64.44	20.19	68.00	52.00–80.00	0.964	< 0.001
FIQR physical	17.95	7.07	19.00	13.00–23.00	0.959	< 0.001
FIQR overall health	12.37	5.65	13.00	8.00–17.00	0.938	< 0.001
FIQR symptoms	34.13	9.41	35.50	28.00–41.00	0.962	< 0.001
PSD	19.77	6.10	20.00	16.00–24.00	0.985	< 0.001
WPI	11.31	4.60	11.00	8.00–15.00	0.975	< 0.001
SSS	8.46	2.53	9.00	7.00–10.00	0.946	< 0.001
FASmod	26.84	7.39	28.00	22.00–32.00	0.972	< 0.001

SD  standard deviation; IQR  interquartile range; BMI  Body Mass Index; FIQR  Revised Fibromyalgia Impact Questionnaire; PSD  PolySymptomatic Distress scale; WPI  Widespread Pain Index; SSS  Symptom Severity Scale; FASmod modified Fibromyalgia Assessment Status

### Seasonal analysis

Of the 2614 patients assessed upon entry into the IFR, 550 were evaluated in autumn, 1140 in winter, 586 in spring, and 338 in summer. Clinimetric indices stratified by season are presented in Table 2.

**Table 2:** Descriptive statistics of clinimetric instruments used to assess fibromyalgia severity across different seasons of the year

Clinimetric tool	Season	*n* patients	Mean	SD	Median	IQR range
PSD	Autumn	550	20.17	5.99	21.00	16.00–25.00
	Summer	338	19.16	5.95	19.00	15.00–23.80
	Winter	1140	19.80	6.22	20.00	16.00–24.30
	Spring	586	19.69	6.05	20.00	16.00–24.00
WPI	Autumn	550	11.70	4.43	12.00	9.00–15.00
	Summer	338	10.84	4.53	11.00	7.25–14.80
	Winter	1140	11.30	4.74	11.00	8.00–15.00
	Spring	586	11.25	4.51	11.00	8.00–15.00
SSS	Autumn	550	8.47	2.46	9.00	7.00–10.00
	Summer	338	8.32	2.38	8.00	7.00–10.00
	Winter	1140	8.50	2.61	9.00	7.00–11.00
	Spring	586	8.44	2.51	9.00	7.00–10.00
FIQR	Autumn	550	65.31	18.73	68.00	52.00–80.00
	Summer	338	63.07	21.28	66.00	49.25–79.00
	Winter	1140	64.49	20.72	67.00	52.00–80.00
	Spring	586	64.31	19.82	68.00	52.00–80.00
FIQR physical	Autumn	550	17.97	6.68	19.00	13.00–23.00
	Summer	338	17.48	7.54	19.00	12.00–24.00
	Winter	1140	18.01	7.19	19.00	13.00–24.00
	Spring	586	18.10	6.91	19.00	13.00–23.00
FIQR overall health	Autumn	550	12.54	5.50	14.00	9.00–17.00
	Summer	338	12.23	5.78	13.00	8.00–17.00
	Winter	1140	12.46	5.62	13.00	9.00–17.00
	Spring	586	12.13	5.78	13.00	8.00–17.00
FIQR symptoms	Autumn	550	34.80	8.72	36.00	29.00–41.00
	Summer	338	33.36	9.87	35.00	28.00–41.00
	Winter	1140	34.06	9.77	35.00	28.00–41.00
	Spring	586	34.08	9.04	35.00	29.00–41.00
FASmod	Autumn	550	27.57	7.04	28.00	23.00–33.00
	Summer	338	26.20	7.46	27.00	22.00–32.00
	Winter	1140	26.61	7.68	27.00	22.00–32.00
	Spring	586	26.99	7.07	27.50	23.00–32.00

SD  standard deviation; IQR  interquartile range; PSD  PolySymptomatic Distress scale; WPI  Widespread Pain Index; SSS  Symptom Severity Scale; FIQR  Revised Fibromyalgia Impact Questionnaire; FASmod  modified Fibromyalgia Assessment Status

Table 3 reports the results of the Kruskal–Wallis test applied to clinimetric indices according to seasonal categorization, both for the entire cohort and stratified by sex. Statistically significant differences were observed in the total cohort for both WPI (*p* = 0.042) and FASmod (*p* = 0.037). When analyzing only the female subgroup, differences became more significant for both WPI (*p* = 0.016) and FASmod (*p* = 0.018). No significant seasonal differences were observed within the male subgroup.

**Table 3:** Comparison (Kruskal–Wallis test) of clinimetric variables used to assess fibromyalgia severity across different seasons of the year. Analyses conducted on the entire sample and stratified by sex

		All the patients		Female patients		Male patients	
Clinimetric variables	df	*χ*^2^	*p*	*χ*^2^	*p*	*χ*^2^	*p*
PSD	3	6.57	0.087	7.60	0.055	1.05	0.787
WPI	3	8.18	0.042*	10.33	0.016*	1.07	0.783
SSS	3	3.73	0.293	2.50	0.474	2.69	0.442
FIQR	3	1.50	0.683	0.65	0.884	0.67	0.879
FIQR physical	3	1.56	0.669	0.90	0.825	0.99	0.803
FIQR overall health	3	1.40	0.706	0.80	0.848	0.37	0.945
FIQR symptoms	3	3.50	0.321	2.37	0.499	1.15	0.765
FASmod	3	8.49	0.037*	10.09	0.018*	0.76	0.859

df  degrees of freedom; PSD  PolySymptomatic Distress scale; WPI  Widespread Pain Index; SSS  Symptom Severity Scale; FIQR  Revised Fibromyalgia Impact Questionnaire; FASmod  modified Fibromyalgia Assessment Status; *  significant test

### Pairwise seasonal comparisons

Considering the entire cohort without sex stratification, pairwise comparisons using the DSCF test revealed significant differences between autumn and summer for both WPI (*W* = −4.009; *p* = 0.024) and FASmod (*W* = −3.80; *p* = 0.037). No other significant differences were detected in other seasonal pairwise comparisons for WPI, FASmod (Table 4), or for the remaining clinimetric indices of disease severity (Table 5).

**Table 4:** Pairwise comparisons between individual seasons (performed using the Dwass-Steel-Critchlow-Fligner test) for clinimetric variables that showed significant differences in the Kruskal–Wallis test

	WPI		FASmod	
Pairwise comparisons between seasons	*W*	*p*	*W*	*p*
Autumn—summer	−4.009	0.024*	−3.80	0.037*
Autumn—winter	−2.379	0.333	−3.26	0.097
Autumn—spring	−2.557	0.270	−2.05	0.466
Summer—winter	2.319	0.356	1.33	0.782
Summer—spring	1.860	0.553	2.12	0.437
Winter—spring	−0.447	0.989	1.10	0.866

WPI  Widespread Pain Index; FASmod  modified Fibromyalgia Assessment Status; *  significant test

**Table 5:** Pairwise comparisons between individual seasons (performed using the Dwass-Steel-Critchlow-Fligner test) for clinimetric variables that did not show significant differences in the Kruskal–Wallis test

	PSD			SSS		FIQR total		FIQR physical		FIQR overall health		FIQR symptoms	
Pairwise comparisons between seasons		*W*	*p*	*W*	*p*	*W*	*p*	*W*	*p*	*W*	*p*	*W*	*p*
Autumn–summer	−3.57		0.056	−1.68	0.635	−1.64	0.651	−0.80	0.942	−0.87	0.926	−2.63	0.244
Autumn–winter	−1.68		0.632	1.01	0.889	−0.43	0.990	0.86	0.929	−0.19	0.999	−1.46	0.729
Autumn–spring	−2.08		0.454	−0.15	1.000	−0.79	0.943	0.88	0.925	−1.40	0.752	−1.71	0.620
Summer–winter	2.53		0.277	2.65	0.239	1.46	0.730	1.51	0.707	0.79	0.943	1.56	0.688
Summer–spring	1.80		0.581	1.57	0.683	0.99	0.896	1.48	0.721	−0.33	0.995	1.19	0.833
Winter–spring	−0.66		0.966	−1.21	0.828	−0.43	0.990	0.07	1.000	−1.37	0.765	−0.38	0.993

PSD  PolySymptomatic Distress scale; SSS  Symptom Severity Scale; FIQR  Revised Fibromyalgia Impact Questionnaire

### Analysis of individual FIQR items related to non-painful symptoms

Analysis of individual FIQR items related to non-painful symptoms across the entire cohort, according to seasonality, revealed no statistically significant differences in fatigue (FIQR item 12, *p* = 0.444), sleep quality (item 15, *p* = 0.150), degree of depression (item 16, *p* = 0.334), memory problems (item 17, *p* = 0.832), or anxiety (item 18, *p* = 0.213).

## Discussion

This study demonstrates the presence of significant differences in fibromyalgia pain severity depending on the season of year in which patients are assessed. These differences specifically concern widespread pain-related symptoms, which are significantly more severe in autumn compared to summer, whereas non-pain symptoms characteristic of fibromyalgia do not appear to be affected. These conclusions apply to the female population with fibromyalgia, as male patients were not impacted. The differences emerge in relation to two patient-reported measures that are central to the assessment of widespread pain in individuals with fibromyalgia, namely, the WPI and the FASmod. Although the FASmod includes two items assessing fatigue and non-restorative sleep, it is predominantly focused on evaluating widespread pain, as it incorporates a front/back body manikin that closely mirrors the one used in the WPI.

To the best of our knowledge, this is the first study to identify a circannual variation in the severity of widespread pain among individuals with fibromyalgia. It is noteworthy that only widespread pain appears to be influenced by seasonality, whereas other symptoms—which typically have a substantial impact on the quality of life of patients with fibromyalgia, including non-restorative sleep, memory problems, depressive symptoms, and anxiety—do not show significant seasonal variation in their assessment.

Our findings may have significant clinical implications. The first relates to patient education, namely, informing female patients that during the autumn season the symptom of “widespread pain” may worsen, while no significant changes are expected for other symptoms. The second concerns therapeutic strategies, suggesting that treatment approaches could be adjusted seasonally to prevent autumn exacerbations.

The observed differences in widespread pain, which concern only female patients, can be explained by the complex interplay of genetic, hormonal, and sociocultural mechanisms that differentiate males and females and render females more pain-sensitive [19]. Our findings are consistent with the extensive literature on sex-as-a-biological-variable in pain research.

Specifically concerning seasonality and fibromyalgia, the number of studies conducted to date remains limited. Contrary to previous publications, we were able to document a seasonal worsening of pain—measured as widespread pain using the WPI—with higher severity observed in autumn compared to summer.

Recently, Castel and colleagues did not report any significant seasonal differences in pain symptoms [12]. Similarly, a prior study by Hawley and Wolfe in 1994, conducted on 2523 patients with rheumatologic conditions, found no substantial seasonal fluctuations in pain symptoms among fibromyalgia patients [20]. However, unlike these two studies, which employed unidimensional pain scales—namely, the Numeric Rating Scale (NRS) and the Visual Analog Scale (VAS)—our study utilizes a different metric, focusing specifically on the extent of pain distribution, as assessed by the WPI. Currently, the use of disease-specific assessment scales is recognized as the reference clinimetric model, particularly when evaluating a complex condition such as fibromyalgia.

The potential influence of seasonality on musculoskeletal pain perception has been acknowledged for centuries. The findings on this topic are not always consistent or conclusive. A significant portion of symptom variability appears to be attributable to weather conditions, which, however, affect individual rheumatic diseases differently [21].

Most of the existing research has focused on low back pain, for which seasonal fluctuations have been demonstrated, with a higher annual prevalence seemingly concentrated in the winter months. In Italy—the country where the present study was conducted—infodemiological data reveal a seasonal peak in Google searches related to low back pain during the winter season [22].

Some of the hypotheses proposed to explain the seasonal variation in low back pain may also help to interpret the findings of the present study. These explanations are largely grounded in the biopsychosocial model. During the summer, increased sun exposure leads to elevated levels of serotonin and vitamin D, both of which are known to positively influence pain perception. Summer also promotes physical activity, which plays a key role in reducing musculoskeletal pain and contributes to greater caloric expenditure. Additionally, summer is generally associated with holiday time, offering individuals more opportunities for recreational activities [23]. Conversely, all of these protective factors tend to diminish in the autumn season.

Seasonal differences do not appear to be solely attributable to climatic factors, which have not been shown to significantly impact symptom severity in female patients with fibromyalgia [24].

The discussion becomes more intricate when taking into account certain “inflammatory” characteristics of fibromyalgia. It has been well established that specific inflammatory mediators are present at elevated concentrations in the serum of patients with fibromyalgia [25]. Recent evidence has also demonstrated the presence of immunoglobulin G in patients with fibromyalgia, which can transfer pain hypersensitivity to mice, with the site of this transfer being the dorsal root ganglia [26]. For certain inflammatory diseases, such as Crohn’s disease and ulcerative colitis, a seasonal pattern has been demonstrated, with higher endoscopic activity observed in autumn for Crohn’s disease and in spring for ulcerative colitis. Seasonal variations have also been reported in systemic inflammatory markers, such as C-reactive protein and erythrocyte sedimentation rate [27]. While such findings cannot be directly extrapolated to fibromyalgia, they underscore the intricate interplay between biological, psychological, and social factors in complex disorders. This complexity is particularly pronounced in fibromyalgia, a condition for which a definitive pathophysiological model has yet to be established.

This study is not without limitations, which must be taken into account when interpreting the findings. The primary limitation of this study lies in the unequal sample sizes across different seasons. Nevertheless, the large overall sample size provided by the IFR contributes to the robustness of the study findings, allowing for further stratification of the population by sex.

A second limitation concerns the stratification by sex itself. It should be noted that there was a clear predominance of female participants, reflecting the prevalence of fibromyalgia in the general population. The underrepresentation of male patients may have affected the statistical power of the analysis in this subgroup.

A third limitation, somewhat related to the first one, is that the seasonal grouping was based on the date of inclusion in the IFR. In this sense, healthcare utilization may have been influenced by external factors unrelated to seasonality (e.g., appointment availability, waiting lists, holiday periods), which could have introduced a selection bias.

In conclusion, widespread pain, as measured by WPI, exhibits significant seasonal fluctuations in the female population affected by fibromyalgia, appearing more severe in autumn compared to summer. The seasonal variation of widespread pain should be taken into account in patient education and in the development of personalized treatment strategies.

## Data Availability

Data are available upon reasonable request to the corresponding author.
